# Reconstruction of Craniomaxillofacial Bone Defects Using Tissue-Engineering Strategies with Injectable and Non-Injectable Scaffolds

**DOI:** 10.3390/jfb8040049

**Published:** 2017-11-20

**Authors:** Bipin Gaihre, Suren Uswatta, Ambalangodage C. Jayasuriya

**Affiliations:** 1 Department of Bioengineering, College of Engineering, University of Toledo, Toledo, OH 43607, USA; Bipin.Gaihre@rockets.utoledo.edu; 2 Department of Orthopedic Surgery, College of Medicine and Life Sciences, University of Toledo, Toledo, OH 43614, USA; Suren.Uswatta@rockets.utoledo.edu

**Keywords:** craniofacial reconstruction, bone, stem cells, scaffolds, injectable, growth factors, biomaterials

## Abstract

Engineering craniofacial bone tissues is challenging due to their complex structures. Current standard autografts and allografts have many drawbacks for craniofacial bone tissue reconstruction; including donor site morbidity and the ability to reinstate the aesthetic characteristics of the host tissue. To overcome these problems; tissue engineering and regenerative medicine strategies have been developed as a potential way to reconstruct damaged bone tissue. Different types of new biomaterials; including natural polymers; synthetic polymers and bioceramics; have emerged to treat these damaged craniofacial bone tissues in the form of injectable and non-injectable scaffolds; which are examined in this review. Injectable scaffolds can be considered a better approach to craniofacial tissue engineering as they can be inserted with minimally invasive surgery; thus protecting the aesthetic characteristics. In this review; we also focus on recent research innovations with different types of stem-cell sources harvested from oral tissue and growth factors used to develop craniofacial bone tissue-engineering strategies.

## 1. Introduction

Craniofacial bone tissue engineering becomes challenging due to the presence of complex physiological structures such as sensory transduction pathways and sophisticated tissue structures, including sensory organs, facial skeletal features, cartilage and blood vessels [1,2,3]. Moreover, clinicians have to control bacterial contamination in highly susceptible areas, including the oral and nasal regions [2]. The necessity for craniofacial tissue engineering arises mainly due to congenital malformations and trauma through sports and various other accidents [3]. The regeneration of facial skeletal tissues must consider ways to ensure the reinstatement of aesthetic characteristics. Additionally, reconstruction should give sufficient mechanical strength to protect internal organs and support movement due to speech and masticatory functions [4,5].

According to the US National Institutes of Health, $697 million was spent to repair the cranial grafts of 12,700 children [6]. A review by Zuk et al. states that, each year, 75% of birth defects reported in the US are cranial defects, which affect more than 225,000 children [7]. Another report claims that more than 6% of a total of 1,600,000 bone grafts implanted annually are craniofacial bone grafts [8]. According to this statistical data, it is clear that with the growth of population in the US, more cranial grafts will be required and the cost of cranial reconstruction will be higher.

Oral-tissue injuries are often considered as a subset of craniofacial injuries, as those injuries are always associated with injuries in craniofacial regions. Statistics shows that more than 20 million people in the US are missing all of their teeth and more than 100 million are missing 11 to 15 teeth [9]. This tooth loss is occurring as a result of trauma, orthodontic treatment, or in the majority of cases due to dental caries or periodontal disease [9,10]. Periodontal disease is a later form of gingivitis that mainly affects the gum and bone that surrounds and supports the teeth. Statistics for periodontal disease indicate that 47.2% of adults in the US, aged 30 years or more, suffer some form of periodontal disease [9]. The annual cost associated with various forms of dental-related activities, including regular dental checkups along with dental implantations and cosmetic interventions, is about $70 billion for Americans.

Various approaches to the treatment and restoration of craniofacial bone defects exist where the utilization of autografts and allografts are considered the best approach [11,12,13]. However, these strategies are associated with their own drawbacks, including limited availability in case of autografts and potential immunogenic rejection when it comes to the utilization of allografts [14,15,16]. On the other hand, regardless of the cost associated with it, dental implants have been promoted as better replacement strategies [17,18]. The failure of these implants, however, is a limiting factor in their use. Most of these failures are a consequence of the implant characteristics, site of implantation, as well as patient-related factors [19,20]. Tissue engineering has, thus, been found to be a clinically relevant approach for regenerating tissue in craniofacial regions.

Tissue engineering is a multidisciplinary field focused on the development of materials and strategies by merging the principles, methods and knowledge of chemistry, physics, engineering and biology [21,22]. This approach involves three fundamental elements: cells, scaffolds, and cell signaling, which are vital for eliciting the essential response from a designed tissue-engineered system [23,24,25]. This review focuses on the various dental and craniofacial bone-tissue engineering approaches involving injectable and non-injectable scaffolds, concentrating in particular on novel injectable scaffolds.

## 2. Structure of the Craniofacial Bone Tissues

The craniofacial region includes facial skin, muscles, bone, tendons, ligaments, nerves and blood vessels. Craniomaxillofacial bones consist of cranial and facial bones [26,27]. Cranial bones enclose the brain and function mostly to protect it, whereas different facial bones such as the maxillary and mandible act as load-bearing bones for the dental region [27]. Bone consists of collagen fibers which are mineralized by hydroxyapatite (HA) to give a crystal structure and thus to provide mechanical strength. Osteoblasts differentiated from the bone marrow-derived mesenchymal stem cells (BMMSCs) regulate osteoid secretion and bone mineralization. Before osteoid mineralization, 94% of the osteoid is collagen fiber [28]. When the bone forms, osteoid is mineralized by calcium apatite to form a HA-like structure. Once the mineralization is complete, calcified bone is composed of 25% organic matrix, 70% mineral and 5% water [28]. Bones are highly vascularized to provide nutrients and oxygen to the bone cells and to remove debris in the extracellular matrix. Other than osteoblasts, osteocytes and osteoclasts also facilitate bone regeneration and remodeling [29].

The dental structure in the craniofacial region includes both hard and soft tissues. The hard-tissue component of teeth includes three structures as enamel, cementum and dentine, while the soft-tissue component is the pulp cavity [30]. Dental tissue engineering is mostly focused on the dentin and pulp tissues of the teeth. The dentin component of teeth is rich in collagen fibers as well as the HA that is important in the bone bonding of dentin structures. The pulp cavity of the teeth is a highly vascularized component which is rich in blood vessels, nerve fibers and lymph vessels. It consists of specialized odontoblast cells and undifferentiated mesenchymal stem cells which are found to be differentiated into dentin-forming cells in vitro and in vivo [31,32]. Odontoblasts are specialized dentin-forming cells present in the dental pulp which are differentiated from dental pulp stem cells (DPSCs). Periodontal ligament is another important component of dental tissue that connects the cement found in the root region of a tooth to alveolar bone and is the best source of periodontal ligament stem cells (PDLSCs). PDLSCs are studied extensively for their differentiation properties in bone-tissue engineering [33,34,35].

## 3. Stem Cells in Craniofacial Bone-Tissue Engineering

The ability of a stem cell to differentiate into many different cell types offers great potential in regenerative medicine [36,37,38]. Based on the source they originate in, these cells are classified as embryonic stem cells (ESCs) and adult stem cells, with the former extracted from embryos developed from eggs fertilized in vitro and the latter extracted from adult tissue and support to maintain and repair the same tissue [39,40].

Human ESCs (hESCs), human BMMSCs (hBMMSCs), and human umbilical cord-derived mesenchymal stem cells (hUCMSCs) have mostly been studied for craniofacial tissue engineering [39,40,41,42,43]. hESCs are harvested from human embryos 5–7 days old and do not exist in the human body. They fall under the pluripotent stem-cell classification and have an ability to form three main germ layers: endoderm, mesoderm and ectoderm. They possess the highest level of pluripotency and an ability to proliferate rapidly [44]. However, due to controversial issues concerning hESCs and ethical practice, hESCs used in research are somewhat limited.

**Figure 1:**
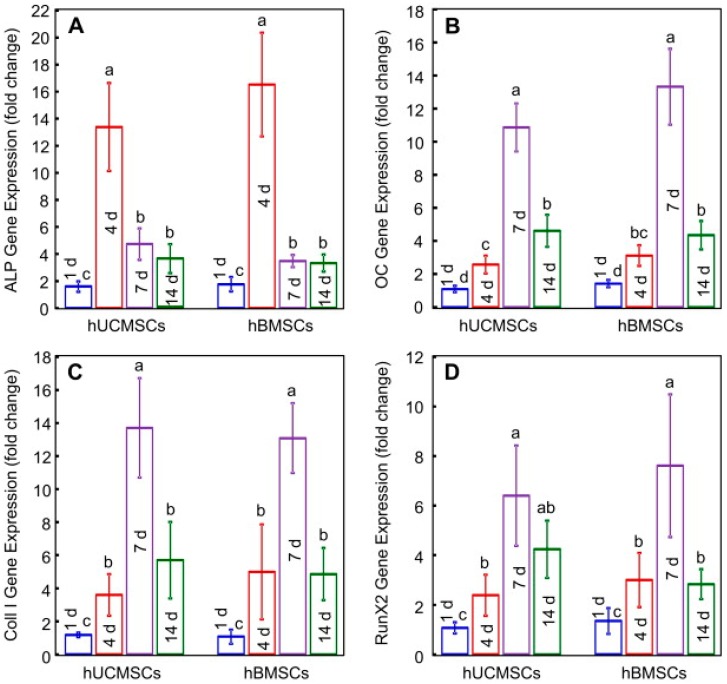
Reverse transcription polymerase chain reaction (RT-PCR) results for the osteogenic differentiation of human umbilical cord-derived mesenchymal stem cells (hUCMSCs) and human bone marrow-derived mesenchymal stem cells (hBMMSCs) on macroporous calcium phosphate cements (CPC): (**A**) alkaline phosphatase (ALP); (**B**) osteocalcin (OCN); (**C**) collagen type 1 (Coll I); and (**D**) runt-related transcription factor 2 (Runx2) gene expressions. The experiments are performed at 1st, 4th, 7th and 14th day. Bars with dissimilar letters indicate significantly different values (*p* < 0.05). This figure is reproduced with the permission from Weir et al. [47]. Copyright Elsevier, 2013.

hBMMSCs and hUCMSCs are harvested from bone marrow and the umbilical cord, respectively, and have been extensively studied in the tissue-engineering field. Both cell lines are multipotent and can be differentiated into osteoblasts, chondrocytes, myoblasts, adipocytes, fibroblasts and nerve tissues [39,45]. hBMMSCs are considered to be the current gold standard cell lines [46]. However, they have certain drawbacks such as an invasive procedure to harvest the cells, a limited number of cells, and lower self-renewal and proliferation capacity due to patient aging and diseases such as arthritis. To avoid these drawbacks, hUCMSCs can be a good replacement. In addition to the advantages of hBMMSCs, hUCMSCs can provide a low-cost source of stem cells, and possess high plasticity and developmental flexibility, minimum immune rejection, and no tumorigenicity [47]. Since the hUCMSC line is harvested from a baby’s umbilical cord, these cells are younger than hBMMSCs. One identified drawback of hUCMSCs is their inability to differentiate spontaneously into osteoblasts, as additional growth factors are required for differentiation as opposed to hBMMSCs, which show characteristics similar to osteoblasts and differentiate quickly. However, Chen et al. [47] experimentally proved that the osteogenic potential of both cell lines are similar over the long-term. Their results show the gene expressions of osteogenic markers such as alkaline phosphatase (ALP), osteocalcin (OCN), collagen type 1 (Coll I), and runt-related transcription factor 2 (Runx 2), are statistically similar with a significance level of 95% (Figure 1).

Several populations of cells, having stem-cell properties, have been extracted from different parts of the tooth, including the pulp, of both exfoliated and adult teeth [48,49]. Periodontal ligament has generic mesenchymal cell properties. DPSCs extracted from adult human dental pulp showed a high proliferation rate in vitro and were developed into dental pulp complex in the in vivo environment [50]. Cavalcanti et al. assessed the odontoblastic differentiation of DPSCs seeded on biodegradable scaffolds studied on a tooth slice model [51]. They were able to demonstrate that their cell-seeded tissue construct model was able to express different odontoblast markers such as dentin matrix protein 1 (DMP-1) and dentin sialo-phosphoprotein (DSPP) confirming the odontoblastic differentiation of DPSCs.

Stem cells from human exfoliated deciduous teeth (SHED) are derived from the disposable deciduous teeth of children which itself is an advantage when compared to other sources of dental stem cells. It has been shown that these cells have a higher proliferation rate than the stem cells from other permanent teeth and possess the ability to develop into dental pulp tissues [52]. Another popular stem cell used in dental tissue engineering is the PDLSC which is extracted from discarded teeth and has the potential to generate the cementum and periodontal ligament-like structure. Studies have shown that these stem cells also have the potential to develop into the osteogenic and adipogenic tissues in vitro, opening up multiple possibilities for tissue engineering from dental-derived stem cells [53].

## 4. Biomaterials for Scaffold Fabrication

Scaffolds are the short-term material framework designed in a specific way to provide an appropriate environment for the proliferation and differentiation of seeded cells, and hence facilitate the development of the desired tissue [23,26,54,55,56]. The design of a scaffold should demonstrate general requirements such as ease of handling, adequate porosity, biodegradability, bioactivity, and appropriate mechanical strength, and should not trigger any immunogenic responses. Thus, the selection of biomaterials always plays an important role in the in vitro and in vivo success of craniofacial tissue-engineered scaffolds.

The biomaterials used for scaffold preparation are natural biopolymers, synthetic polymers, ceramics and composites [56,57,58,59,60,61,62,63]. Natural biopolymers include chitosan [28,60,61,62,63], alginate [46,64,65], cellulose [66,67,68], collagen [33,35,69], hyaluronan [70,71,72], fibrin [73,74,75,76] and silk [77,78]. The most commonly used synthetic polymers for scaffold preparation include poly (L-lactic acid) (PLA) [79,80], poly(lactic-co-glycolic acid) (PLGA) [81,82,83,84], polycaprolactone (PCL) [80,85,86], and poly(propylene fumarate) (PPF) [87,88].

Calcium phosphate cement (CPC), calcium sulfates, bioactive glasses, calcium carbonates and HA are the most used ceramic materials to fabricate scaffolds for bone-tissue regeneration [25,44,47,56,58]. CPC has been used as a scaffold to study the cell adhesion, cell proliferation and differentiation of different types of cells [44,47]. CPC is widely used as a scaffold for cranial defects research. CPC supports resorption by gradually replacing the scaffold area with bone as bone formation progresses. The cell-adhesion properties of CPC can be improved by grafting RGD (Arg–Gly–Asp) peptide motif to it which is identified by cell membranes [53]. Modified CPC scaffolds are used to study the osteogenic capacity of different cell lines such as hBMMSCs, hUCMSCs and hESCs [44,47]. 

**Figure 2:**
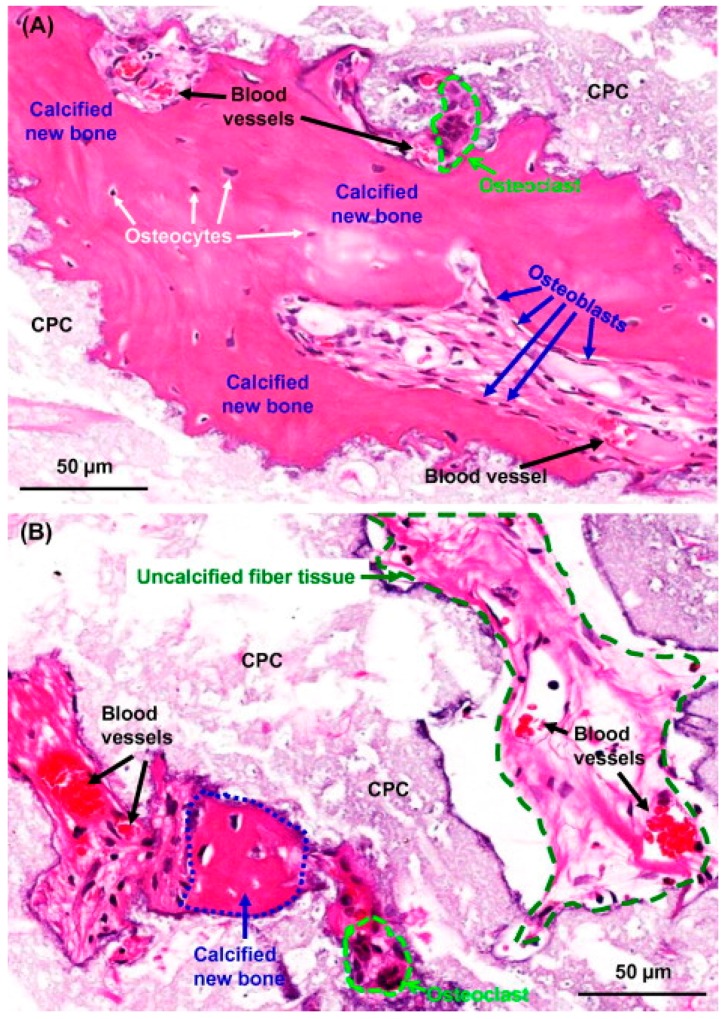
High-magnification hematoxylin and eosin (HE) staining images. (**A**) New bone grew in the interior of the CPC scaffold and was maturing, as indicated by the presence of osteocytes and blood vessels. (**B**) Both calcified new bone and uncalcified new bone matrix were observed. This figure is reproduced with the permission from Weir et al. [44]. Copyright Acta Materialia, 2014.

Figure 2, shows hematoxylin and eosin (HE) staining images where more calcified bone can be observed in CPC scaffolds seeded with hESCs compared to CPC scaffolds without hESCs [44].

## 5. Injectable and Non-Injectable Scaffolds

Both injectable and non-injectable scaffolds have been extensively studied in dental and craniofacial bone-tissue engineering. Recent studies have been focused on the injectable scaffolds as these have potential for limiting the invasive procedures [89,90] and can fit those scaffolds on any defective area regardless of the shape and size of the bone. Some of the major successes in bone grafting and scaffolds are shown in Table 1. These bone grafts and scaffolds are approved by the Food and Drug Administration (FDA) for human use. While some of these grafts and scaffolds such Collagraft, Infuse, have been in the market for a long time, PRO-DENSE and MASTEERGRAFT are relatively newer products available. Table 2 shows some major products and technologies under clinical trial, with trials on some of these already completed.

### 5.1. Injectable Scaffolds

Recent studies have started to focus on using injectable scaffolds as they can be inserted into the defect site with non-invasive surgery [26,71,73,84,98]. Some of the major properties of these scaffolds are highlighted in Table 3. Minimum invasive surgery can prevent infection, morbidity, surgical scars and extensive blood loss [89,90]. Injectable materials must possess mechanical properties and provide structural stability at the defect site. Promising injectable scaffolds should be embodied with growth factors which must be released in a controlled manner to promote cell attachment, proliferation and differentiation. Different types of injectable scaffolds such as microparticles [56,64,67,84,90,99], hydrogel [45,46], nanocomposite films [60,100], nanoparticles [62,101] and membranous scaffolds [102,103] are being developed. Jiang et al. developed biomimetic, membranous, spiral cylindrical scaffold using chitosan-based membrane. In vivo studies showed that these spiral membranous scaffolds were completely infiltrated by bone tissues, which grew along the spiral wall connecting both ends of the defects [66]. Aryaei et al. characterized the mechanical properties of nanocomposite films prepared by dispersing multi-walled carbon nanotubes (MWCNT) on a chitosan matrix [60]. Even though they proved no cytotoxicity of the chitosan/MWCNT composite films during in vitro studies, in vivo studies need to be done on this nanocomposite.

Alginate is one of the most commonly used natural polymer which forms stable hydrogels in the presence of metallic ions. It is a copolymer of mannuronic acid (M unit) and guluronic acid (G unit), which are important in determining the mechanical properties of alginates. Moshaverinia et al. explored the potential of alginate hydrogel microparticles as a medium for the encapsulation of dental-derived mesenchymal stem cells [46]. They observed stem cell-encapsulated alginate microspheres did not affect the stem cell properties, as both cell viability and proliferation were excellent at both early and later stages of culture. The in vitro cell-differentiation study on the alginate-encapsulated periodontal ligament stem cells (PDLSCs) and gingival mesenchymal stem cells (GMSCs) showed the ability of these stem cells to differentiate into adipogenic and osteogenic tissue [46]. The modulation of the biodegradation rate of the scaffold material is always important for tissue-engineering approaches and of high importance, especially when they are used as a cell-delivery vehicle. A study by Moshaverinia et al. has demonstrated that the biodegradation of alginate hydrogel can be controlled by modulating the degree of oxidation of alginate [46]. Another study conducted by them used alginate microspheres embedded with hBMMSCs and with growth factor-recombinant bone morphogenic protein-2 (rhBMP-2) or anti-BMP-2 mAb, separately, on critical size calvarial defects to test whether anti-BMP-2 mAb can influence the BMP-signaling pathway [64]. Their results successfully demonstrated that hBMMSCs encapsulated in alginate microspheres can enhance the osteogenic differentiation of hBMMSCs by activating the BMP-signaling pathway.

Chitosan is widely used to construct scaffolds for bone regeneration [56,61,63,65,66,98,99]. It is the most available biopolymer, second only to cellulose. Chitosan has a hydrophilic surface to promote cell adhesion, proliferation and osteogenic differentiation of MSCs and the formation of bone matrix [56,61,63]. Mechanically, scaffolds prepared only with chitosan fail to provide sufficient structural integrity and support. Therefore, chitosan is cross-linked with sodium tripolyphosphate, glutaraldehyde and genipin [56,98,99,104]. Moreover, due to its lack of solubility at physiological pH, proteolytic enzymes causes pre-systemic metabolism of drugs [63].

Fibrin is a non-reactive, highly elastomeric, less cytotoxic and highly binding homeostatic agent [73,74,75]. Fibrin can be rapidly degraded by cells due to its high biodegradability, and this process of breaking down fibrin is called fibrinolysis [76]. It can be obtained from fibrinogen which is a major structural protein of blood-clotting and conducive to wound healing. Fibrinogen can be polymerized to derive fibrin by employing thrombin to remove its A and B peptides [76]. It has been found that a higher rate of osteoid formation takes place when fibrinogen is injected with aprotinin solution compared to osteoid formation by fibrinogen with saline solution. However, studies have found the mechanical strength of new bone to be inferior when fibrin gel is used [75].

Self-assembling peptide hydrogels have also been explored as injectable scaffolds in the form of gels. These hydrogels, also available under the commercial name of Puramatrix, comprise 16-mer peptide in aqueous solution [45]. The most important property of Puramatrix, that makes it suitable as an injectable gel, is its ability to instantaneously polymerize. This forms a biodegradable scaffold when introduced in the physiological condition. Cavalcanti et al. studied the response of dental pulp stem cells (DPSCs) seeded on Puramatrix and cultured in a tooth slice [45]. Their results showed that the DPSCs are able to survive and proliferate in a 3-D Puramatrix scaffold. The rationale for using a tooth-slice model to culture the seeded stem cells was to assess the impact of dental-derived factors on the odontoblastic differentiation of DPSCs. It was observed that DPSCs seeded on the Puramatrix without a tooth slice did not express markers of odontoblastic differentiation, suggesting dental-derived factors are required for odontoblastic differentiation.

Currently, the regeneration of dental tissues is focused on the application of bio-functional agents on the scaffold material [105,106]. A recent study by Thein-Han et al. explored this approach for dental-tissue engineering [105]. They utilized the excellent in situ hardening property of CPC and significantly improved the cell attachment to CPC by incorporating bio-functional agents, for instance, the RGD (Arg–Gly–Asp) sequence, and assessed the differentiation of hUMSCs. Before studying the cellular response of the scaffold, they looked at the mechanical properties of the modified scaffold. There was no change in the setting time of the CPC injectable as well as no significant change in the flexural strength of the bio-functionalized scaffold material. They observed that the flexural strength of these modified scaffold materials was relatively higher than that of polymeric and hydrogel scaffolds, making them suitable for a load-bearing application. Besides, their study also demonstrated the excellent biocompatibility of these scaffolds and the rapid proliferation of seeded hUMSCs [105].

Hyaluronate is another biopolymer that is used as an injectable scaffold [70,71,72,107]. It is a fluid that is already present in the human body, especially in joints and eyes. It has been used as an injectable scaffold with growth factors to engineer new bone formation. Martinez-Alavarez et al. used injectable hyaluronate hydrogel with HA and BMP-2 to repair the cleft palate of canine models [107]. According to their results, when these gels were injected through minimal pinholes, there were no adverse effects such as the excessive swelling that was reported to occur when using open surgery. They also observed complete cleft closure developed without further surgical scars and also without creating the need for mucoperiosteal flaps.

### 5.2. Non-Injectable Scaffolds

Non-injectable scaffolds are also used in craniofacial tissue engineering [98,108,109]. Major comparison of these scaffolds with injectable scaffolds are underlined in Table 3. These scaffolds are fabricated in the predetermined shape and composition that will provide 3-D support to the cells using different fabrication methods [110,111,112]. Ceramics have mostly been used for this purpose when compared to natural polymers and other biomaterials, because of their suitable porosity and chemical textures which support the differentiation and mineralization of seeded cells [113].

**Figure 3:**
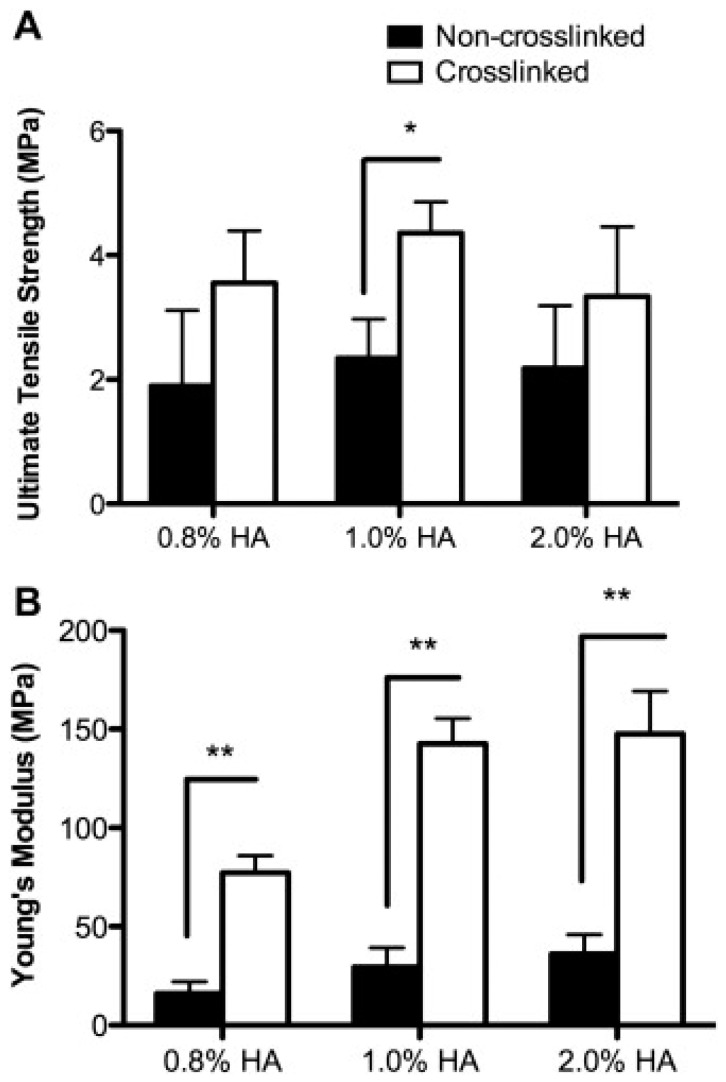
(**A**) Ultimate tensile strength and (**B**) Young’s moduli of non-crosslinked (black bars) 0.1% genipin and crosslinked (white bars) 7% chitosan nanofibers at different concentrations of hydroxyapatite. (* and ** represent statistical significance at *p* < 0.05 and *p* < 0.01). The figure is reproduced with the permission from Lelkes et al. [104]. Copyright Elsevier, 2012.

Frohbergh et al. fabricated electrospun scaffolds for non-weight bearing bones such as cranial bones in order to simulate the role of periosteum [104]. Their scaffolds were made using electrospinning a chitosan solution dispersed with HA nanoparticles. To increase the Young’s modulus of the periosteum, electrospun fibers were crosslinked with genipin, a natural crosslinker (Figure 3). Their final results have shown that these scaffolds can be osteoinductive and can be used to replace the function of the periosteum. Incorporation of HA in the scaffold reduced the Young’s modulus. To avoid this, Frohbergh et al. used nanoHA dispersed on the electrospun fibers. Their results indicated that dispersing HA nanoparticles does not only increase mechanical strength but also supports the function of mineralization [104].

Scaffolds developed with substantial functional microvasculature can be beneficial in establishing the circulation of oxygen, nutrients and growth factors throughout the biomaterial after implantation. Chen et al. prepared macroporous scaffolds using CPC powder (consisting of tetracalcium phosphate and dicalcium phosphate), chitosan liquid and the gas-forming porogen method [109]. Additionally, they bio-functionalized the scaffold by incorporating RGD domains in chitosan. Their results showed that RGD-incorporated scaffolds have a significant increase in the microcapillary-like structures on them compared to CPC without the RGD scaffolds. They concluded that focusing on the formation of prevascularized cement can enhance the acclimation of the scaffolds in an in vivo environment.

Calcium phosphate and chitosan scaffolds with rhBMP-2 can induce bone formation. In a study conducted by Gunzman et al. new bone was observed in defects filled with chitosan/rhBMP-2/calcium phosphate scaffolds compared to no new bone formation observed in rhBMP-2/chitosan or calcium phosphate/chitosan scaffolds [114]. This study also showed that rhBMP-2 and calcium phosphate implanted in a defect without a scaffold degraded after three weeks. This is because growth factors such as recombinant human BMP-2 has a short half-life and is rapidly cleared after the injection. Therefore, these factors must be incorporated in a suitable biopolymer to maintain controlled released, effective local concentration, and long-term bioavailability.

Goudouri et al. studied the perspectives of modified bioactive silicates containing Mg phase as a scaffold for dental tissue engineering [115]. These modified bioactive glass ceramics, termed akermanite, were able to generate the calcium phosphate layer in an in vitro environment. This indicated the ability of these scaffolds to bond to the living bone when implanted into the body [115]. In another study, Gronthos et al. studied the in vivo response of dental pulp stem cells (DPSCs) seeded on the powdered HA and tri-calcium phosphate (TCP) scaffold [49]. The cell biomaterial was pelleted and implanted into immunocompromised mice. Their study was able to generate a structure quite similar to the dental pulp complex and the orientation of collagen fibers in the regenerated tissue complex was quite similar to that in the dental pulp. In a similar study, Zhang et al. used the same scaffold material in a porous form and observed the in vivo response [31]. The porous scaffold was able to induce abundant ingrowth of tissue and vasculature but the regeneration of the dental pulp complex was not observed.

A few natural polymers such as chitosan, collagen, cellulose, alginate and fibrin have also been explored for their potential in dental-tissue engineering, among which the collagen scaffold is the most popular [33,35,69,103]. Cross-linked collagen type-I scaffolds have been studied with DPSCs and it has been shown that these scaffolds were able to induce cellular differentiation and abundant calcification of the developed extracellular matrix in an in vitro environment [31].

## 6. Growth Factors

In order to obtain a desired differentiation and phenotypes to regenerate dental and craniofacial bone tissues, relevant physicochemical signals should be applied to the scaffolds or cements. This can be done through the incorporation of growth factors in the scaffold in different ways, such as encapsulating, immobilizing them by chemical bonding, or simple coating in the scaffold matrix [63,78,79,82,87,94,116]. Once they are incorporated into the scaffolds, they must release and diffuse in the extracellular matrix to give directional and spatial cues to the cells in in vivo conditions. However, there are a number of limitations to using exogenous growth factors such as side effects, high cost, and their functional capacity is also lower compared to growth factors that are already present in the extracellular matrix. Furthermore, the release and delivery kinetics are difficult to control as expected.

**Figure 4:**
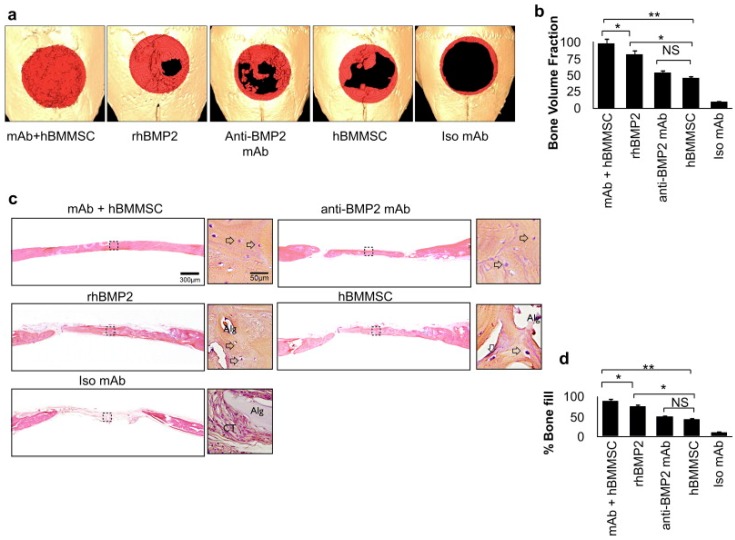
hBMMSCs encapsulated in RGD-alginate microspheres loaded with anti-BMP2 mAb contribute to bone regeneration in a critical-size calvarial defect model. (**a**) Micro-computed tomography (micro-CT) results of bone repair in mouse calvarial defects. Regenerated bone is pseudo-colored red; (**b**) semi-quantitative analysis of bone formation based on micro-CT images; (**c**) microanatomic representation of repair of critical-size defects in the mouse calvaria after 8 weeks of transplantation at high (40×) and low (4×) magnifications stained with HE. Arrows point to osteocytes in lacunae (Alg: unresorbed alginate, CT: connective tissue); (**d**) histomorphometric analysis of calvarial defects showing the relative amount of bone formation in the critical size calvarial defect model. (* and ** represent *p* < 0.05 and *p* < 0.01 respectively). The figure is reproduced with the permission from Zadeh et al. [64]. Copyright Elsevier, 2013.

Large number of studies have shown that rhBMP-2 and rhBMP-7, can induce osteogenesis on large defects in the craniofacial region [64,65,79,87,116,117]. However, there are number of disadvantages attributed to the use of rhBMP-2, such as the requirement for high physiologic doses of rhBMP-2, high cost, and the inability to sustain bioactivity for a long period of time. Furthermore, under in vivo conditions it has been shown that rhBMP-2 can lead to unwanted ectopic bone formation while also stimulating immune reactions [118,119]. Schuette et al. reported a transient autoimmunity induced by both rhBMP-2 and rhBMP-7. They found the prevalence of 18% for the rhBMP-7 autoantibody (aAB) in treated patients, which was much higher than in the case of healthy patients. Similar results were observed for rhBMP-2 with a higher expression of BMP-2 aAB in treated patients [119]. To overcome this issue, Moshaverinia et al. used anti BMP-2 monoclonal antibodies (anti BMP-2 mAb) incorporated into RGD-coupled alginate microspheres [64].Their results showed that anti BMP-2 mAb can capture BMP-2 ligands in the scaffold site and present them to the osteoprogenitor cells. According to their study, the expression of osteogenic markers such as decapentaplegic homolog1 (Smad 1) protein and Runx2 protein is similar to scaffolds transplanted with only anti-BMP-2 mAb and only rhBMP-2. Micro-computed tomography (CT) and histomorphometric analysis showed bone formation at the calvarial defect by the scaffolds with mAb is higher than by the scaffolds with rhBMP 2 (Figure 4).

The insulin-like growth factor-1 (IGF-1) is another growth factor that can simulate the chemotactic movements of osteoblasts cells [99]. IGF-1 can induce the movement of osteoblasts to the defect site to start osteogenesis. Like other growth factors, IGF-1 activity is concentration-dependent and, depending on the concentration gradient, the influence of IGF-1 on osteogenesis changes. Studies have shown that the concentration dependence of these growth factors also depends on the cell-culture system. Detamore et al. reported that IGF-1 at low concentration was more effective in stimulating collagen synthesis from temeporo mandibular joint (TMJ) cells when grown in 3-D scaffolds compared to the cells growing in a monolayer where a higher concentration of IGF-1 was required. Their study also established IGF-1 as the best growth factor compared to the basic fibroblast growth factor (bFGF) and transforming growth factor (TGF) for TMJ regeneration [120]. The release of the growth factor from the scaffolds depends on the pH and temperature of the medium, degree of crosslinking, and method of incorporation (encapsulation or coating). Mantripragada et al. showed that, due to the swelling properties of chitosan, at higher pH microparticles become globular and decrease the pore sizes on surface, thus reducing the release of encapsulated IGF-1 [99].

Growth factors such as the vascular endothelial growth factor (VEGF) and platelets-derived growth factor (PDGF) can also influence the angiogenesis process [82,121,122,123]. Hu et al. showed that VEGF and PDGF support the formation of bloods vessels [118].They also showed that the expression of these growth factors can be improved by blood-derived platelet-rich plasma (PRP). Liu et al. used PRP and hESCs-seeded CPC scaffolds to test if they can influence greater bone formation and higher blood-vessel density compared to the CPC scaffolds seeded with only hESCs [47]. Their results showed that the formation of bone in the PRP–hESC scaffolds is 49% higher than in the control samples. It can thus be inferred that PRP can induce blood-vessel formation and thereby increase bone formation simultaneously.

## 7. Future Directions

Most of the studies in craniofacial tissue engineering have been focused more on non-injectable scaffolds, which are designed in a predefined shape and require complicated surgical techniques for implantation. While with the advent and studies on 3-D printing technologies this approach looks promising, the structural complexity of these tissues make this approach challenging at the same time. However, within the past few years a number of studies have been undertaken on the development of injectable scaffolds mainly involving natural polymers. The future of craniofacial tissue engineering with injectable scaffolds looks promising. Although recent success has been demonstrated in the delivery of cells and bioactive components with natural biomaterial scaffolds, more research is needed to improve the mechanical properties of these scaffolds, especially if they are to be used in craniofacial and dental hard tissue engineering. Besides, more intensive study is required into the development of injectable scaffolds with synthetic materials which are more advantageous than natural materials in terms of their mechanical properties, availability, and material properties.

## 8. Conclusions

This review paper aims to discuss recent advances in craniofacial tissue engineering. Craniofacial tissue is the region that requires the highest consideration in tissue engineering due to associated aesthetic characteristics. For the successful regeneration of complex tissue structures and the restoration of aesthetic characteristics, numerous biomaterials and scaffolds have been used. Before deciding on biomaterials and scaffolds, we need a good understanding of the complex anatomical structures of craniofacial tissue. The Introduction of this review emphasizes the necessity for the tissue-engineering approach to solve existing craniofacial tissue-related issues. We focused on cell lines that can be used to accelerate the healing of the wound and expedite tissue regeneration. Choosing the optimum biomaterial is another challenging task that has to consider rigorously the structure of the host tissue, biocompatibility and the facilitation of the seeded cell lines. We discussed various scaffolds used in recent studies. These scaffolds are incorporated with different growth factors to support the differentiation of cells into target cells. The different types of growth factor and their roles in regenerating tissue are also discussed. Craniofacial regeneration is possible with injectable and non-injectable scaffold systems, with the injectable system being preferable.

## Figures and Tables

**Table 1:** List of a few bone grafts/scaffolds approved by Food and Drug Administration (FDA).

Name	Materials	Biologic Agent	Approved Use	References
Collagraft	60% Hydroxyapatite 40% Tricalcium phosphate and fibrillar collagen	X	Long bone fractures	[91]
PRO-DENSE	75% Calcium sulfate 25% Calcium phosphate	X	Open bone voids/gaps of extremities and pelvis (no structural support)	[92]
MASTERGRAFT family of products				
a) MASTERGRAFT Granules and Mini Granules	85% Beta tricalcium phosphate	X	Fusion of multiple level of posterolateral spine	[93]
b) MASTERGRAFT Putty	15% Hydroxyapatite Above cement composition with type I bovine collagen	X		
Norian	Calcium phosphate Polylactide/glycolide copolymer Sodium hyaluronate	X	Bony voids/gaps of extremities and pelvis (not intrinsic to stability of structure)	[94]
BoneSource	Calcium phosphate salts Sodium phosphate buffer	X	Filling burr hole and facial skeleton augmentation	[95]
Mimix	Calcium phosphate Sodium citrate dehydrate Anhydrous citric acid solution	X	Filling burr hole, craniotomy defects, smoothing facial skeleton contour abnormalities	[96]
INFUSE	Type I bovine collagen sponge	Recombinant bone morphogenic protein-2 (rhBMP-2)	Tibia fracture and non-union and lower spine fusion	[97]
GEM 125	Beta-tricalcium phosphate	Recombinant platelet derived growth factor (rhPDGF-BB)	Periodontal defects	[97]
Osteogenic protein-1	Type I bovine collagen powder	Bone morphogenic protein-7 (BMP-7)	Long bone non-union	[97]

**Table 2:** Some scaffolds/technologies under clinical trials (Accessed from clinical trial resource of US National library of medicine, www.clinicaltrials.gov).

Name	Materials	Biologic Agent	Use (Current Status)
Gene-activated matrix (Nucleostim)	Collagen hydroxyapatite	DNA plasmids with gene encoding vascular endothelial growth factor (VEGF-A165)	Regeneration of bone tissues in maxillofacial area (enrolling)
3-D printing technology	Plastic 3-D templates for bending the titanium reconstruction plates	X	Stabilization of facial fractures (proposed)
Sepax tool	Decellularized bone matrix (DBM) injectable	Mononucleotide autologous stem cells	Non-union/delayed fractures (unknown)
Bone void filler	Starch hydroxyapatite	X	Open fracture of foot (completed)

‘X’ indicates no biological agent.

**Table 3:** Comparison of major features of injectable and non-injectable scaffolds.

Injectable Scaffolds	Non-Injectable Scaffolds
Developed in a way that can be easily injected with minimally invasive techniques and can form a stable structure within the body.	Developed in a predefined shape and structure depending on the nature of defect and need to be implanted into defect site through surgeries.
Can conform to different shape independent of defect structure after injection.	Prior knowledge of the shape and size of the defect to be implanted is needed. The defects with irregular shape and location, as in the craniofacial region, could be difficult to fill with these scaffolds.
Minimize patient discomfort, risks of infection and scar formation.	The proper distribution of bioactive agents and the seeded cells in the scaffold matrix is problematic due to their morphology and structure. This limits their applicability in in vivo conditions due to lower cell ingrowth and tissue formation.
Can provide a more homogenous distribution of bioactive agents and, hence, better therapeutic effects can be achieved as these agents are introduced in a solution or suspension form.	The mechanical strength of these scaffolds is higher compared to that of injectable scaffolds. Different materials can be used, and better scaffold fabrication techniques can be used to achieve higher strength and other desired physical properties.
Injectable polymeric hydrogels lack sufficient mechanical strength, limiting their application in load-bearing applications compared to injectable bio-ceramic paste, which possesses higher mechanical strength.	These scaffolds are crosslinked prior to implantation, making them stable and resistant to hydrolytic degradation.
Solidification of these scaffolds in an in vivo condition takes place through ceramic setting, thermal crosslinking and gelation, and self-assembly.	Synthetic polymers, metals and bio-ceramics are mostly used to fabricate these scaffolds.
Injectable scaffolds in the form of microparticles, pastes and gels are studied extensively.	The degradation of these scaffolds is slower when compared to injectable scaffolds which are mostly developed using natural polymers.
